# Analysis of colostrum IgA against bacteria involved in neonatal infections

**DOI:** 10.1590/S1679-45082017AO3958

**Published:** 2017

**Authors:** Elizabeth Moreira Dias, Denise Bertulucci Rocha Rodrigues, Vinícius Rangel Geraldo-Martins, Ruchele Dias Nogueira

**Affiliations:** 1 Universidade de Uberaba, Uberaba, MG, Brazil.

**Keywords:** Enterobacteriaceae, Staphylococcus aureus, Colostrum, Immunoglobulin A, Infection, Enterobacteriaceae, Staphylococcus aureus, Colostro, Imunoglobulina A, Infecção

## Abstract

**Objective:**

To describe e compare the specificity of IgA antibodies against bacteria extract of *Klebsiella pneumoniae* , *Staphylococcus aureus* , *Escherichia coli* , and *Salmonella enteritidis* .

**Methods:**

Colostrum samples were aseptically collected in the first 12 hours after C-section delivery. The specificity of IgA against bacteria extracts was analyzed by the Western blot.

**Results:**

The findings showed proteins of high molecular weight frequently detectable in the samples. *S. aureus* was the most frequently found bacterium in the samples (p<0.05). Approximately 93.8, 56.3, 62.5 and 60.4% of samples presented IgA reactive to *S. aureus* , *K. pneumoniae* , *S. enteritidis,* and *E. coli,* respectively. Roughly 40% of samples showed no IgA reactive to *K. pneumoniae, S. enteritidis* and *E. coli* .

**Conclusion:**

Clinical evidence of the importance of breastfeeding for the immune protection of neonates was consistent with the observed immunological findings, since most samples showed IgA reactive against the species tested. The application and development of immunotherapies during pregnancy, focused on frequently detected antigens, could be an important tool to enhance the presence of IgA in colostrum.

## RESUMO

**Objetivo:**

Descrever e comparar a especificidade de anticorpos IgA de amostras de colostro contra extratos bacterianos de *Klebsiella pneumoniae* , *Staphylococcus aureus* , *Escherichia coli* e *Salmonella enteritidis* .

**Métodos:**

As amostras de colostro foram coletadas assepticamente nas primeiras 12 horas após o nascimento por cesariana. A especificidade de IgA contra extratos de bactérias foi analisada por Western blot.

**Resultados:**

Os achados mostraram proteínas de alto peso molecular frequentemente detectáveis nas amostras. *S. aureus* foi a bactéria mais encontrada nas amostras (p<0,05). Cerca de 93,8, 56,3, 62,5 e 60,4% das amostras apresentaram IgA reativa a *S. aureus* , *K. pneumoniae* , *S. enteritidis* e *E. coli* , respectivamente. Aproximadamente 40% das amostras não apresentaram IgA reativa contra *K. pneumoniae* , *S. enteritidis* e *E. coli.*

**Conclusão:**

A evidência clínica da importância da amamentação para proteção imunológica ao recém-nascido foi consistente com os achados imunológicos observados, uma vez que a maioria das amostras mostrou IgA reativa contra as espécies testadas. A aplicação e o desenvolvimento de imunoterapias durante a gestação, focada nos antígenos frequentemente detectados, poderiam ser importantes instrumentos para aumentar a presença de IgA no colostro.

## INTRODUCTION

Neonatal bacterial infection remains a major cause of morbidity and mortality during the neonatal period. According to the World Health Organization, 6.3 million children under 5 years old died in 2013, and 45% of them during the neonatal period. ^(^ ^1^ ^)^ In Brazil, about 60% of infant mortality is mainly due to bacterial infections. ^(^ ^2^ ^)^

The greater susceptibility to neonatal bacterial infection is explained by the relative immunological immaturity of newborns, ^(^ ^3^ ^)^ which is a direct consequence of immune adjustment during the transitional period, from intra- to extra-uterine life. ^(^ ^4^ ^)^ Ontogeny of the immune system begins in the embryo and continues during fetal life, but it is completed only a few years after birth. ^(^ ^5^ ^)^ However, hospital, maternal and obstetric practices may contribute to the occurrence of bacterial contamination that may result in gastrointestinal and respiratory infections, ^(^ ^6^ ^)^ neonatal sepsis and systemic involvement. ^(^ ^2^ ^)^

Neonatal sepsis, the third most common cause of death in early life, results in half a million deaths each year. ^(^ ^7^ ^,^ ^8^ ^)^ The pathogens most commonly isolated and involved in neonatal infections are *Klebsiella pneumoniae* , *Escherichia coli, Salmonella enteritidis* and *Staphylococcus aureus* . ^(^ ^8^ ^)^

Although *E. coli* colonizes the gastrointestinal tract of the neonate within a few hours of life and develops a mutualistic relation with the host, ^(^ ^9^ ^)^ this species is the one most frequently involved in neonatal sepsis. ^(^ ^10^ ^,^ ^11^ ^)^ Some serotypes of *E. coli,* such as enteropathogenic, enterohaemorrhagic, enteroaggregative and enterotoxigenic, have been reported as the main cause of diarrhea in children under 1 year of age. ^(^ ^12^ ^)^ Also O6 *E. coli* serotype was detected in many cases of neonatal meningitis ^(^ ^13^ ^)^ and sepsis. ^(^ ^14^ ^)^ Another relevant bacterium associated with neonatal gastrointestinal infections is *S. enteritidis* , which usually appears after the first week of life, and causes acute gastroenteritis and thus serious complications to the newborn, such as sepsis and/or meningitis. ^(^ ^15^ ^)^

Also, *S. aureus* has been linked to various infections during the neonatal period, such as late-onset neonatal sepsis, ^(^ ^8^ ^)^ impetigo, ^(^ ^16^ ^)^ arthritis and osteomyelitis. ^(^ ^17^ ^)^ Virulence antigens of *S. aureus* are predominantly related to bacterial surface, such as capsular polysaccharides, teichoic acid, peptidoglycans, adhesins, protein A, and toxins. ^(^ ^18^ ^)^ *Klebsiella pneumoniae* is an opportunistic pathogen that causes pneumonia, bacteremia and urinary tract infections ^(^ ^19^ ^)^ and has been reported as a common agent in cases of neonatal sepsis. ^(^ ^20^ ^)^ It is also associated with high mortality, often through strains multiresistant to antibiotics associated to the production of beta-lactamase. ^(^ ^20^ ^)^

After birth, with the interruption of IgG transfer via the umbilical cord, the mother is able to offer to the newborn another form of passive protection, represented by breastfeeding, which has indisputable protective attributes associated to reduction of the risk of neonatal infection ^(^ ^3^ ^,^ ^21^ ^)^ because it contains several immune components such as secretory IgA (IgAS). ^(^ ^22^ ^)^ The presence of IgAS represents the first line of defense of the mucous membranes, conferring protection against infections and coating mucosal surfaces, preventing the adhesion and invasion of microorganisms in the tissues. ^(^ ^3^ ^,^ ^21^ ^,^ ^23^ ^)^

Although exclusive breastfeeding is recommended and practiced, some newborns that are breastfeeding can develop bacterial infection during the neonatal period. There is evidence that children, although being breastfed, can develop diarrhea by *Campylobacter* due to a lack of specific antibodies against virulence antigens for this bacterium in colostrum. ^(^ ^23^ ^)^ Thus, it is necessary to determine, in samples of colostrum, the presence and specificity of IgA against bacteria commonly involved in neonatal infections, such as *S. aureus, K. pneumoniae, S. enteritidis* and *E. coli* .

## OBJECTIVE

To describe e compare the specificity of IgA antibodies against bacteria extract of *Klebsiella pneumoniae* , *Staphylococcus aureus* , *Escherichia coli* , and *Salmonella enteritidis* .

## METHODS

A total of 48 mothers were enrolled in this study upon consent. The Ethical Committee approved this study CAAE: 02166713.4.0000.5145. Only healthy mothers, 12 hours after delivery, were included in standard collection. Information on maternal and gestational background was obtained through interviews with the mothers. Samples of colostrum were collected by manual expression into sterile polypropylene Falcon tubes. After collection, the maternal samples were transported in ice to the laboratory, centrifuged at 1,300g for 7 minutes to remove lipid components and stored at -80°C until use.

### *Western blotting* of colostrum IgA against bacteria

Extracts of *S. aureus* (ATCC 25923), *K. pneumoniae* (ATCC 13883), *S. enteritidis* (ATCC 13076) and *E. coli* (ATCC 11303) were obtained from fresh culture as previously described. ^(^ ^20^ ^)^ Seventeen micrograms of extracts were separated by 6%-SDS-page and transferred to nitrocellulose membranes. The membranes were incubated with colostrum (1:1,000) samples. After washing, they received a solution of antibody HRP- goat anti-human IgA (Sigma) revealed by the ECL system (Amersham Biosciences Little Chalfont, Buckinghamshire, United Kingdom) and exposed in biofilm for five minutes.

The developed X-ray films were scanned in a scanning densitometer (Bio-Rad GS-700 Imaging Densitometer) and the images were evaluated with ImageQuant Software (Amersham Biosciences) to analyze patterns of antigen recognition, including the number and intensity of reactive bands. Signals were converted to absolute counts by comparison with the standards on the same membrane. Failure to detect a signal was recorded as zero. A film blank value was subtracted from the value of the reactive band.

Some membranes were incubated with blocking buffer without samples to obtain negative control. As positive control, membranes were incubated with a saliva sample whose pattern of reaction with antigen extracts had been previously measured. The reactive bands represent the presence of IgA specific to proteins separated by the SDS page from each bacterium extract.

### Statistical analysis

The mean number of IgA bands and densitometry values of reactive Ags were determined and compared between the bacteria by Analysis of Variance (ANOVA). The frequencies of positive IgA reactive to antigens were assessed by χ ^2^ test. The correlations between IgA antibodies and specific to antigens were tested by Pearson analysis. A p value <0.05 was considered statistically significant.

## RESULTS

The mothers were healthy and no complication during or after delivery was reported. Their mean age was 25.2±3.3 years. There were no differences in racial profile (p>0.05). All babies were born full term (>37 weeks of gestation). No associations were found between immunoglobulin levels and racial, maternal age, type of birth and socioeconomic data (Pearson, p>0.05). There were also no statistically significant differences between immunoglobulin levels and types of delivery: cesarean or vaginal (p>0.05).

### Specificity of IgA response against bacterial extracts

**Figure 1:**
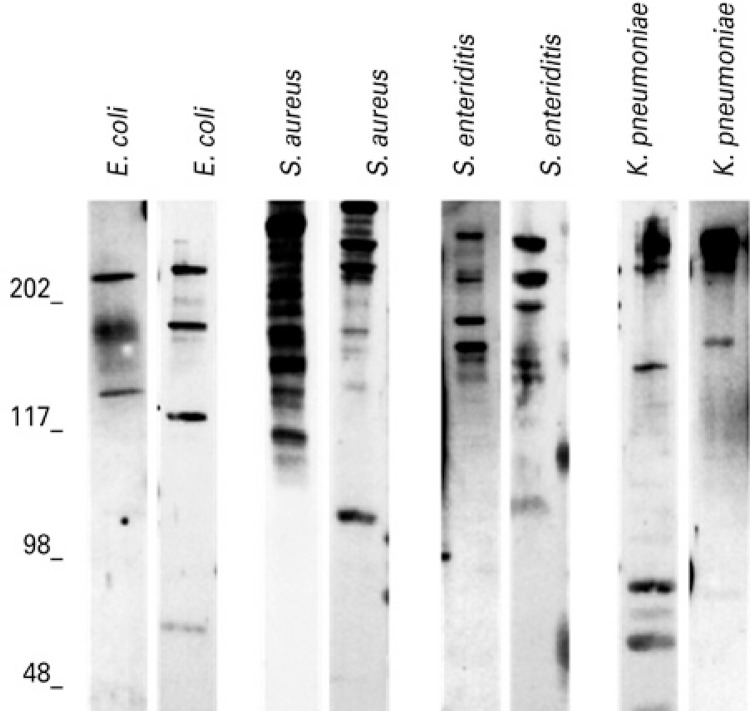
Patterns of IgA reactive against antigens from *Staphylococcus aureus* , *Klebsiella pneumoniae* , *Salmonella enteritidis* and *Escherichia coli* in samples of colostrum. Identities of antigen extracts are above each lane. Standard molecular sizes (kDa) are indicated to the left of the immunoblots

Examples of immunoassays of samples with positive IgA response against the bacterial antigens are represented in figure 1 . The frequencies of samples with positive (with at least one detectable band) and negative responses to the four strains are represented in table 1 . The majority of samples showed reactive IgA against *S. aureus* , followed by *S. enteritidis* , *E. coli* and *K. pneumoniae* ( Table 1 ). The number of samples with positive response to *S. aureus* was statistically higher than for other bacteria ( Table 1 ; p<0.05; q>15.00).

**Table 1:** Intensity and frequency of IgA reactive to *Staphylococcus aureus* , *Klebsiella pneumoniae* , *Salmonella enteritidis* and *Escherichia coli* and antigens detected in colostrum samples

Bacterial extracts and molecular weight (kDa)	Numbers of samples with response		Mean of total intensity of colostrum IgA reactivity ± SD

	Positive n (%)	Negative n (%)	
*Staphylococcus aureus*	45 (93.8) ^*†‡^	3 (6.2) ^*†‡^	410.7±186.3 ^§¶||^
230	26 (57.8)		48.5±41.9
221	18 (40.0)		33.6±41.6
208	25 (55.6)		46.7±42.2
185	17 (37.8)		31.7±41.2
*Salmonella enteritidis*	30 (62.5) ^‡^	18 (37.5) ^‡^	217.8±186.3 ^§¶||^
244	16 (53.4)		31.6±30.3
220	16 (53.4)		30.2±29.7
176	14 (46.7)		27.0±29.4
144	15 (50.0)		28.4±29.6
*Klebsiella pneumoniae*	27 (56.3) ^†^	21 (43.7) ^†^	133.2±101.9 ^¶#^
244	10 (37.0)		16.7±22.2
229	7 (25.9)		12.0±20.7
203	7 (25.9)		10.9±19.6
194	7 (25.9)		12.5±21.9
73	8 (29.6)		13.9±22.4
46	8 (29.6)		13.3±20.9
*Escherichia coli*	29 (60.4) ^*^	19 (39.6) ^*^	251.8±129.9 ^||#^
238	12 (41.4)		27.7±33.6
216	15 (51.7)		34.6±34.0
202	15 (51.7)		32.7±23.8
162	13 (44.8)		30.0±33.9

^*^ χ ^2^: p=0.001, q=15.10; ^†^ χ ^2^: p=0.001, q=18.00; ^‡^ χ ^2^: p=0.001, q=17.10; ^§^ ANOVA: p<0.001; ^¶^ ANOVA: p<0.001; ^||^ ANOVA: p<0.05; ^#^ ANOVA: p<0.05.

SD: standard deviation.

### Complexity of the IgA response against bacterial antigens

**Figure 2:**
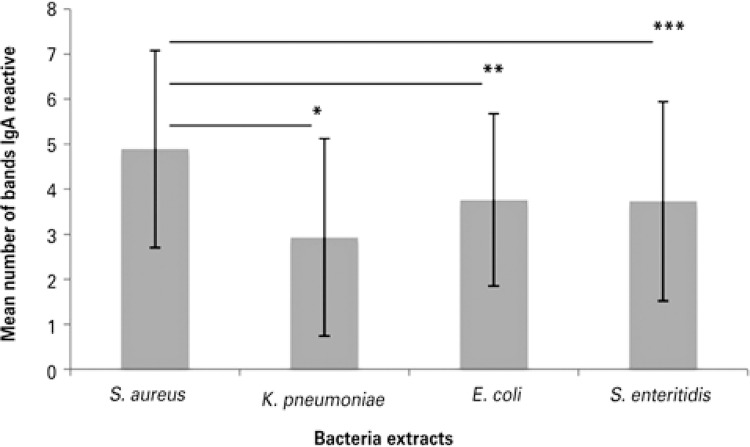
Mean numbers of bands of IgA reactive with the bacterial antigens of *Staphylococcus aureus* , *Escherichia coli* , *Klebsiella pneumoniae* and *Salmonella enteritidis* in colostrum

The number of IgA bands reactive against *S. aureus* ranged from 2 to 11 (mean: 4.89±2.19); to *K. pneumoniae* , from 1 to 11 (mean: 2.93±2.19); to *S. enteritidis* , from 1 to 11 (mean: 3.73±2.21); and against *E. coli* , from 1 to 8 (mean: 3.76±1.911). Mean number of IgA-reactive bands to microbial antigens from *S. aureus* , *E. coli* , *K. pneumoniae* and *S. enteritidis* are represented in figure 2 . The mean number of bands reactive to *S. aureus* was elevated and there were statistically significant differences in comparison to other bacteria ( Figure 2 ; p<0.05). There were no differences in the mean number of bands between *K. pneumoniae* , *S. enteritidis* and *E. coli* (p>0.05).

*p=0.02; **p=0.02; ***p=0.04.

### IgA specific against the bacterial extracts bands separated by SDS-PAGE

**Figure 3:**
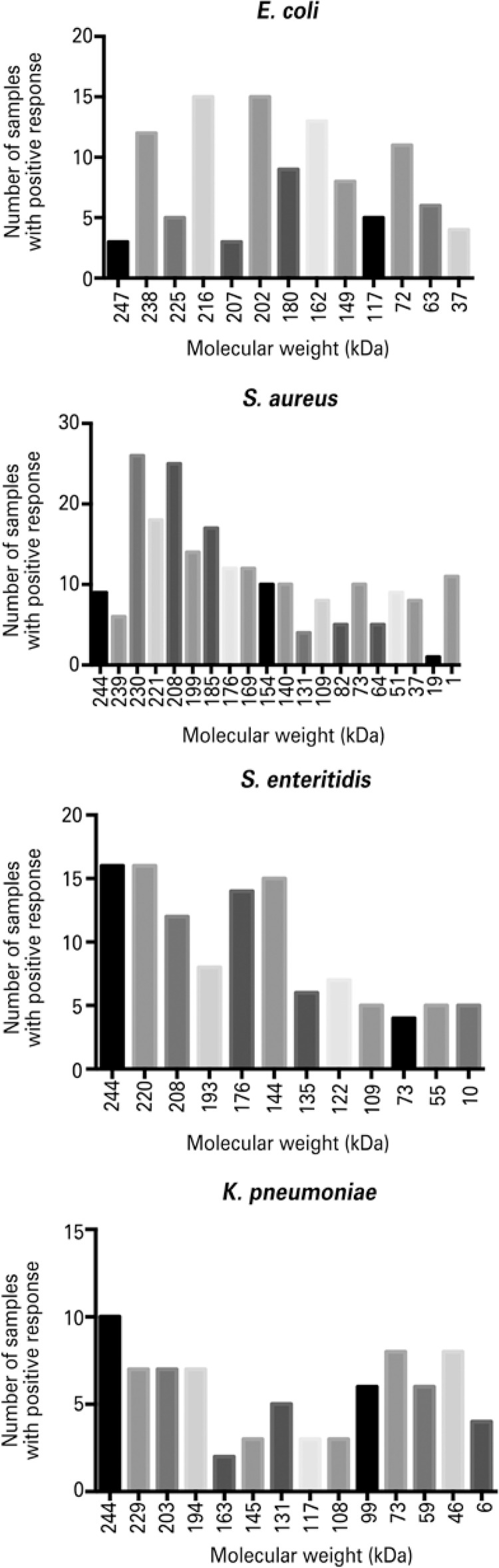
Number of samples with IgA reactive to specific bacterial antigens and their molecular weights (kDa)

IgA bands recognized and detected in bacterial extracts were analyzed, and their molecular weight (kDa) was calculated by the equation obtained by the analysis of the molecular weight standard bands. The number of samples with positive IgA to antigens and their respective molecular weight with *S. aureus, K. pneumoniae* , *S. enteritidis* and *E. coli* are represented in figure 3 . There was a great variation in the bands recognized by IgA for each bacterial extract, and the majority of them showed a high molecular weight ranging between 247-109kDa. Approximately 59 bands of different molecular weights were detected. A 244KDa band was common in samples of *S. aureus* , *K. pneumoniae* and *S. enteritidis* but there was no correlation between them (p>0.05, r<0.28).

The most detected frequencies of samples with positive response to antigens are represented in table 1 . The IgA response against *S. aureus* showed the greatest diversity; more than 20 types of different bands were detected (p<0.05). The reactivity of IgA against *S. aureus* showed a prevalence of bands with high molecular weight (p<0.05, Figure 3 ). Over 55% of samples with positive response to *S. aureus* showed IgA reactive to 230kDa and 208kDa. Twelve different bands were found among samples with positive IgA to *S. enteritidis* (n=30). Bands of 244 and 220kDa were recognized in 53.4% of samples and were statistically more detected than the others ( Table 1 , p<0.05).

The analysis of IgA against *K. pneumoniae* revealed 14 distinct bands ( Figure 3 and Table 1 ). The frequency of positive response against the most detected bands ( Table 1 ) did not differ (p>0.05). The immunoblots of IgA against *E. coli* showed 13 different bands ( Figure 3 and Table 1 ), but there were no differences in the frequency of IgA response between these bands (p>0.05).

There were variations in the intensities of IgA antibody reactions with the recognized bands among children in both groups. Table 1 shows the sums of intensities of IgA reactions with all bands detected for each species (total intensities). In general, the intensity of IgA response was higher to *S. aureus* than other bacteria (p<0.05). In addition, the total intensity of IgA response to *E. coli* was decreased in comparison to *K. pneumoniae* (p<0.05).

## DISCUSSION

The majority of colostrum samples had IgA reactive to bacterial antigens, thus corroborating several studies that emphasize the importance of breast milk, which provides protection against infection by *Haemophilus influenzae* and *Escherichia coli* , ^(^ ^21^ ^)^ and enteric infections caused by *E. coli* , *Vibrio cholerae* , *Campylobacter, Shigella* spp and *Giardia lamblia* . ^(^ ^24^ ^-^ ^26^ ^)^ Thus, breastfeeding can protect neonates against the oral invasion of a large number of microorganisms. ^(^ ^21^ ^)^

The majority of samples (94%) had antibodies IgA reactive against *S. aureus* accompanied by intense and complex responses, since the number of reactive bands was higher than for other bacteria. This high frequency of reactive IgA probably occurred because this bacterium is part of the normal human microbiota in various regions of the human body, which does not exclude the possibility of this microorganism causing disease under immunosuppression, or when epithelial barriers are violated, which can be limited to the mucosal surface or spread throughout the body. ^(^ ^9^ ^)^

Although the majority of samples had reactive IgA against these bacteria, about 40% of samples did not present IgA reactive to *K. pneumoniae* , *S. enteritidis* or *E. coli* , which suggests that some newborns may develop infections from these microorganisms even though they are breastfed. One of the reasons suggested to explain breastfed children with diarrhea by *Campylobacter* is a lack of specific antibodies against common antigens of virulence of *Campylobacter* in samples of colostrum. ^(^ ^23^ ^)^ Thus the importance of not only of studying the presence of IgA against bacteria, but also of evaluating and identifying immunodominant antigens of these species in the natural immune response.

The reasons for lower or higher detection of antibodies in the samples may be associated to the antigenic stimulation. Thus, it is necessary to implement a strategic stimulation to enhance the immune protection of newborns in their neonatal period against such microorganisms, and increase immunogenic antigens, which occurs in meningococcal infection. Prenatal women immunization, with a single dose of meningococcal vaccine, results in an increase in specific IgG levels in the newborn serum for 2 to 3 months after birth, and an increase of the specific IgA levels to the microorganism in the milk for at least 6 months. ^(^ ^23^ ^)^

The results showed a great diversity of the antigen species tested, with a predominance of high molecular weight protein. The most common bands can be related to the pathogenic action and/or antigenic stimulation of these bacteria, such as 230 and 289kDa *S. aureus* ; and the four most prevalent bands of *S. enteritidis* and *E. coli* are shown in table 1 . *K. pneumoniae* showed a great variability in IgA response, but did not show a specific pattern of response. The literature provides some information about several antigens of these bacteria that are involved in their pathogenic capacities, but little is known about the high molecular weight antigens widely recognized by the samples of this study.

An antigen of 94kDa called “intimin” and other antigens recognized by IgA of 70, 80 and 110kDa were associated in the injury process “attaching and effacing” by enteropathogenic *E. coli* in human milk. ^(^ ^6^ ^)^ Also, Rck is a 17KDa outer membrane protein, expressed in *E. coli* and *S. enteritidis* , which inhibits complementary pathways, thereby preventing opsonization through this route. ^(^ ^27^ ^)^ No sample showed IgA reactive to intimin (94kDa) and 17kDa band, but about 38 and 17% of samples responded to a band of 72 and 117kDa, respectively, probably the same as previously reported. ^(^ ^6^ ^)^ *K. pneumoniae* antigens of 35 and 36kDa opsonizing induce antibodies ^(^ ^28^ ^)^ and were recognized by 29% of the present study samples.

The 106kDa antigen (fibronectin type A) is one of the most important molecules involved in the adhesion in the early stages of infection by *S. aureus* . ^(^ ^29^ ^)^ In addition to those, protein constituents of *S. aureus* membrane of 30 and 36kDa may play an important role in infections caused by this bacterium. ^(^ ^30^ ^)^ These antigens were detected in 17% of samples with positive responses.

## CONCLUSION

The majority of colostrum samples can protect newborns against neonatal bacterial infection. It is important to understand how those antibodies provided by breastfeeding could help the mucosal immune system against the colonization and the challenge offered by those species, in order to develop prevention strategies to avoid such infections. The natural response to bacterial extracts tested shows a promising way for the development of vaccines containing antigens that could be applied during pregnancy, increasing the specific IgA levels in colostrum.
